# Pre-operative predictive factors of residual varus on the mechanical axis after Oxford unicompartmental knee arthroplasty

**DOI:** 10.3389/fsurg.2022.1054351

**Published:** 2023-01-09

**Authors:** Songjie Ji, Ye Huang, Yixin Zhou, Chao Wang, Xiaokai Wang, Chaoyi Ma, Xu Jiang

**Affiliations:** ^1^ Department of Orthopedic Surgery, Beijing Jishuitan Hospital, Fourth Clinical College of Peking University, Beijing, China; ^2^ Department of Statistics, Beijing Research Institute of Traumatology and Orthopedics, Beijing, China; ^3^ Department of Radiology, Beijing Jishuitan Hospital, Fourth Clinical College of Peking University, Beijing, China

**Keywords:** lower limb alignment, predictor, unicompartemtal knee arthroplasty, moblie-bearing, biplanar radiograph

## Abstract

**Background:**

Residual varus after Oxford unicompartmental knee arthroplasty (UKA) happens frequently. This study aims to evaluate the pre-operative contributing factors of residual varus.

**Methods:**

A total of 1,002 knees (880 patients, 201 patients were male, and 679 were female) underwent Oxford UKA in the Orthopedic Surgery Department of the Beijing Jishuitan Hospital from March 2018 to April 2021. The mean age of the patient was 64.7 ± 7.7 years. To assess residual varus, the full-length lower extremity is placed upright for EOS imaging, with the knee fully extended. The angle of post-operative residual varus was measured as described by Noyes *et al*. Of the knees studied, they were either categorized into an under-corrected group (post-operative Noyes angle >5°) or a corrected group (post-operative Noyes angle ≤5°). Age, gender, body mass index (BMI), range of motion (ROM), Clinical American Knee Society Score (Clinical AKSS), and Function American Knee Society Score (Function AKSS) were compared. The following additional parameters were measured: pre-operative Noyes angle, lateral distal femoral angle (LDFA), medial proximal tibial angle (MPTA), the posterior slope of the proximal tibia angle (PPTA), joint line converge angle (JLCA), and fixed flexion deformity (FFD).

**Results:**

There was no statistically significant difference between the two groups in regards to gender (*p* = 0.428), surgical leg (*p* = 0.937), age (*p* = 0.851), BMI (*p* = 0.064), pre-operative Clinical AKSS (*p* = 0.206) and Function AKSS (*p* = 0.100). However, pre-operative ROM statistically differed between the two groups (*p* < 0.001). The contributing factors of post-operative residual varus were determined to be the following parameters: pre-operative MPTA (*p* < 0.001, OR = 4.522, 95% CI: 2.927–6.984), pre-operative Noyes (*p* < 0.001, OR = 3.262, 95% CI: 1.802–5.907) and pre-operative FFD (*p* = 0.007, OR = 1.862, 95% CI: 1.182–2.934). The effects of pre-operative LDFA (*p* = 0.146), JLCA (*p* = 0.942), and pre-operative PPTA (*p* = 0.899) on the post-operative mechanical axis did not show statistical significance.

**Conclusions:**

Patients with severe pre-operative varus, particularly varus deformity mainly from the tibial side or pre-operative FFD, are more prone to get extremity mechanical axis residual varus after UKA with Oxford.

## Background

Unicompartmental knee arthroplasty (UKA) is an effective treatment for patients with osteoarthritis (OA), particularly for those with anteromedial osteoarthritis (AMOA) of the knee or localized necrosis of the femoral condyle (1). UKA is less invasive, and patients exhibit a faster recovery time while obtaining better post-operative clinical function (2, 3) compared to total knee arthroplasty (TKA). It has previously been reported that UKA is a more suitable technique for patients of Asian decent, as their lifestyle requires deeper knee flexion than TKA (2). However, multiple research groups and systematic reviews have demonstrated that UKA could lead to a higher revision rate than TKA (3, 4). Many studies have suggested that this may result from an unsatisfactory position of the components. Poor component position, malalignment of the lower limb mechanical axis (correlating with point contact between components), and contact stress have been identified as the major contributing factors to poor clinical outcomes, early polyethylene wear, as well as the high revision rate (5, 6). Overcorrection of the mechanical axis may result in degeneration of the contralateral compartment and lead to premature loosening of the prosthesis (7). Mild under-correction is considered acceptable in the field. Some researchers have suggested that residual post-operative alignment (1°– 4° of varus) is associated with the most optimal functional outcomes after medial UKA (8, 9). A consequence of excessive residual varus alignment, which can lead to UKA failure from polyethylene wear, is increased compartment force by overloading medially (6, 8).

There are two UKA prosthesis options commonly used clinically: mobile-bearing and fixed-bearing. While each type has its advantages and disadvantages (10), fully congruent mobile-bearing UKA (also known as Oxford UKA) appears to be an attractive alternative to fixed-bearing UKA in young and active patients (11). The mobile-bearing Oxford UKA is considered more difficult to ensure postoperative alignment than the fixed-bearing, as it is performed when the knee is in a flexion position (12). Few study on the predictive factors related to the changes in the mechanical axis after this procedure. This study aims to analyze the unsatisfactory mechanical axis cases of the Oxford UKA and discuss the pre-operative factors that may affect postoperative mechanical axis correction.

## Material and methods

### Data source

This study obtained written informed consent from participants or their guardians and was approved by the Beijing Jishuitan Hospital Institutional Review Board for retrospective data analysis. Between March 2018 and April 2021, 1,002 knees (880 patients, including 201 males and 679 females) underwent the Oxford UKA procedure using a single-compartment prosthesis (Zimmer Biomet, Warsaw, IN, United States) with the Microplasty instrumentation. All surgeries occurred at the Beijing Jishuitan Hospital in the Orthopedic Surgery Department. The patient's mean age was 64.7 ± 7.7 years. The inclusion criteria for patients treated with UKA were as follows: ① diagnosed with OA. At the same time, pre-operative weight-bearing x-ray demonstrated single-compartment lesions on the medial knee joint, and the lesions are limited to the anterior and medial tibial plateau. Suppose The lateral compartment is suspected to be involved. That is, the x-ray grade of the lateral platform is grade 1. This case needs to undergo an MRI of the knee joint to confirm no partial loss of lateral cartilage; ② active mobility is greater than 90° by physical examinations; ③ the patients with good condition for anterior cruciate ligament, and MRI was performed to check it if necessary; ④ pre-operative full-length x-ray imaging in the upright standing position of lower extremities showed varus less than 15° (using the Noyes et al. method); ⑤ pre-operative fixed flexion deformity (FFD) is less than 15° (using the Paley et al*.* method). Our inclusion criteria follow Oxford's recommendations (12). The following data was collected from the patients: age, gender, body mass index (BMI), pre-operative knee joint range of motion (ROM), pre-operative knee pain, and functional score (American Knee Society Score, AKSS) (13). ROM was measured by physical examination. A questionnaire survey measured AKSS.

### Surgical procedure

All surgeries were performed using the Microplasty instrumentation for the Oxford unicompartmental knee system (Zimmer Biomet, Warsaw, IN, United States). The whole surgical process is completed under the instruction of Oxford (12). The goal of the procedure was to establish a slight under-correction within the range of 1°–5° of varus to avoid degenerative progression on the lateral compartment (14, 15). It should be emphasized that the medial collateral ligament (MCL) was carefully protected, and there were no cases where an extensive MCL release was performed.

### Evaluation indicators

In preparation for the UKA procedure, all patients underwent a complete body examination using low-dose radiation EOS imaging (EOS Imaging, Paris, France) (16). A low-dose biplanar radiograph, EOS (EOS-Imaging, Paris, France), is an emerging technique to assess a three-dimensional (3D) measurement for the lower limb alignments (17). Simultaneous biplanar x-ray imaging of the EOS system, similar to dual ﬂuoroscope imaging (18), provides an avenue to accurately determine the position of the UKA components and the lower limb extremity mechanical axis in functional positions. As weight-bearing status signiﬁcantly affects the lower limb mechanical axis. Standing upright has been suggested as a criterion for quantitative 3D lower limb alignment (19). The EOS 3D matching and digitization technique can accurately reconstruct the *in-vivo* 3D UKA component position and lower limb mechanical axis in the standing position (20).

**Figure 1:**
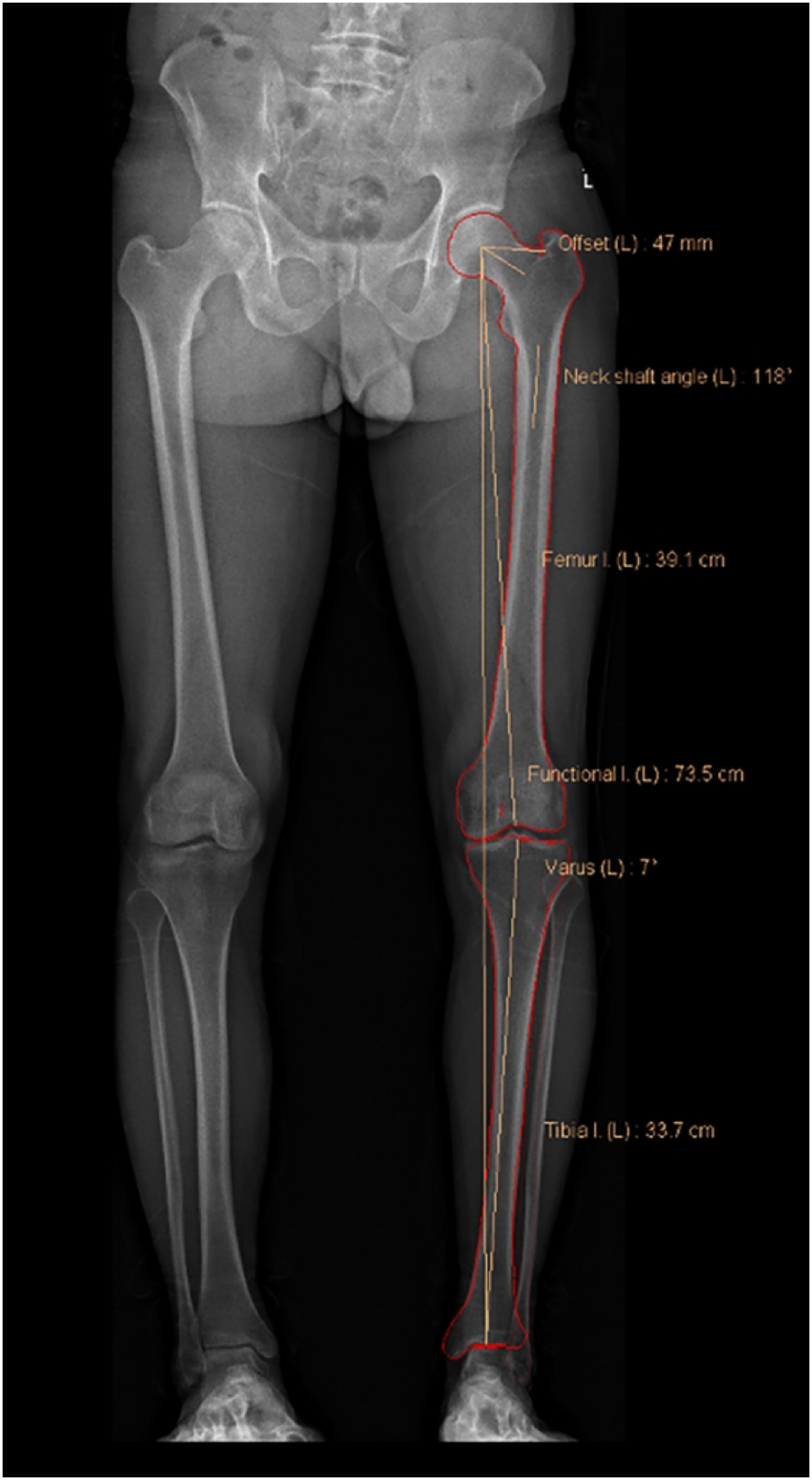
In the coronal plane, the hip joint center point is the center of the circular femoral head, and the center of the distal femur is the top of the intercondylar notch. The mechanical axis of the femur passes through these two points. The center of the proximal tibia is the center of the tibial spines, and the center of the ankle is the mid-width of the tibia and fibula at the level of the plafond. The mechanical axis of the femur passes through these two points (23). Pre-operative Noyes angle: the angle between the femoral mechanical axis and the tibial mechanical axis. In the EOS software, it is designated as “varus.” In this image, the Noyes angle is 7°.

**Figure 2:**
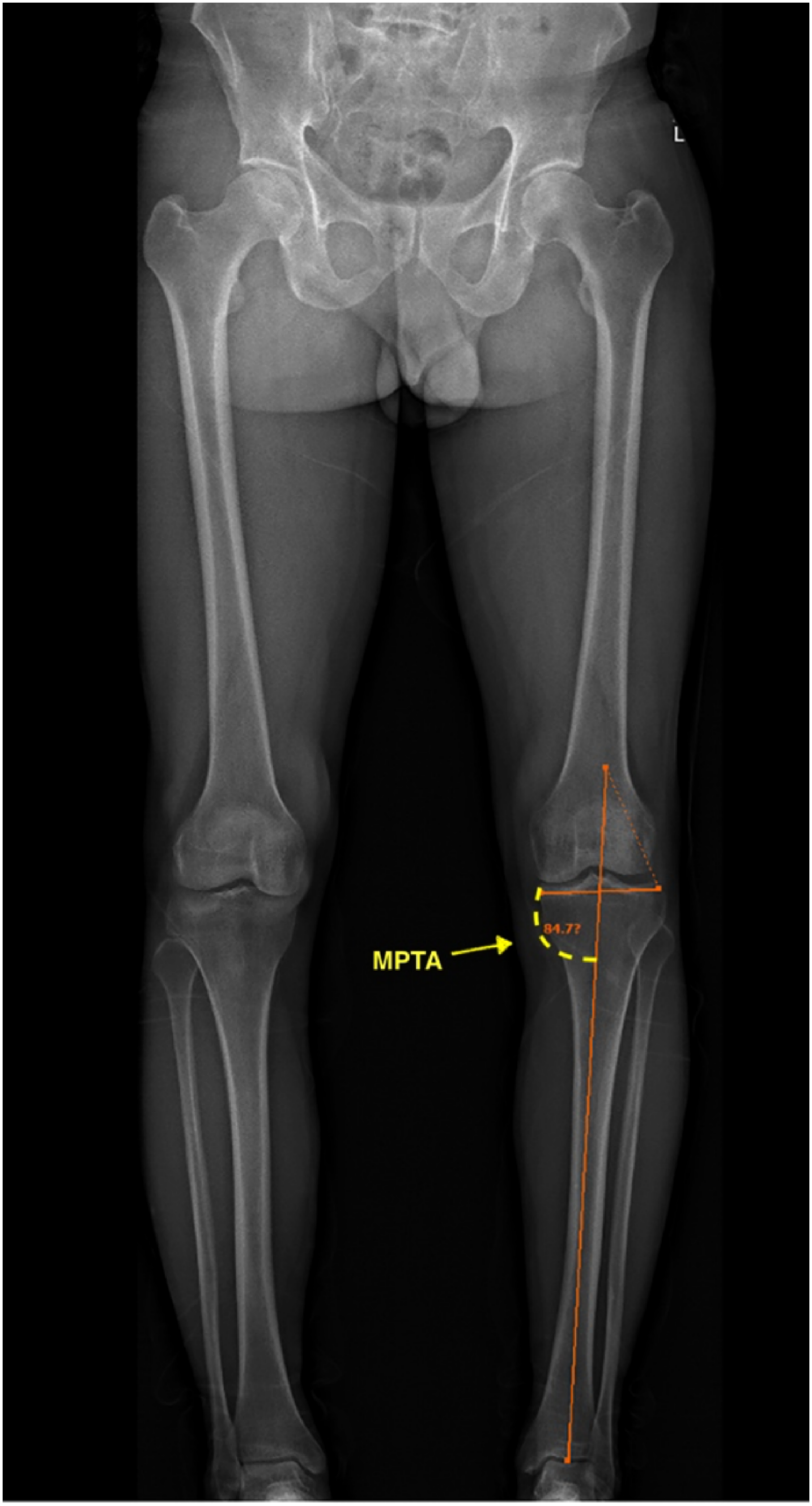
Medial proximal tibial angle (MPTA): the proximal medial angle formed between the tibial mechanical axis and the knee joint line of the tibia in the frontal plane. In this image, the MPTA is 84.7°.

**Figure 3:**
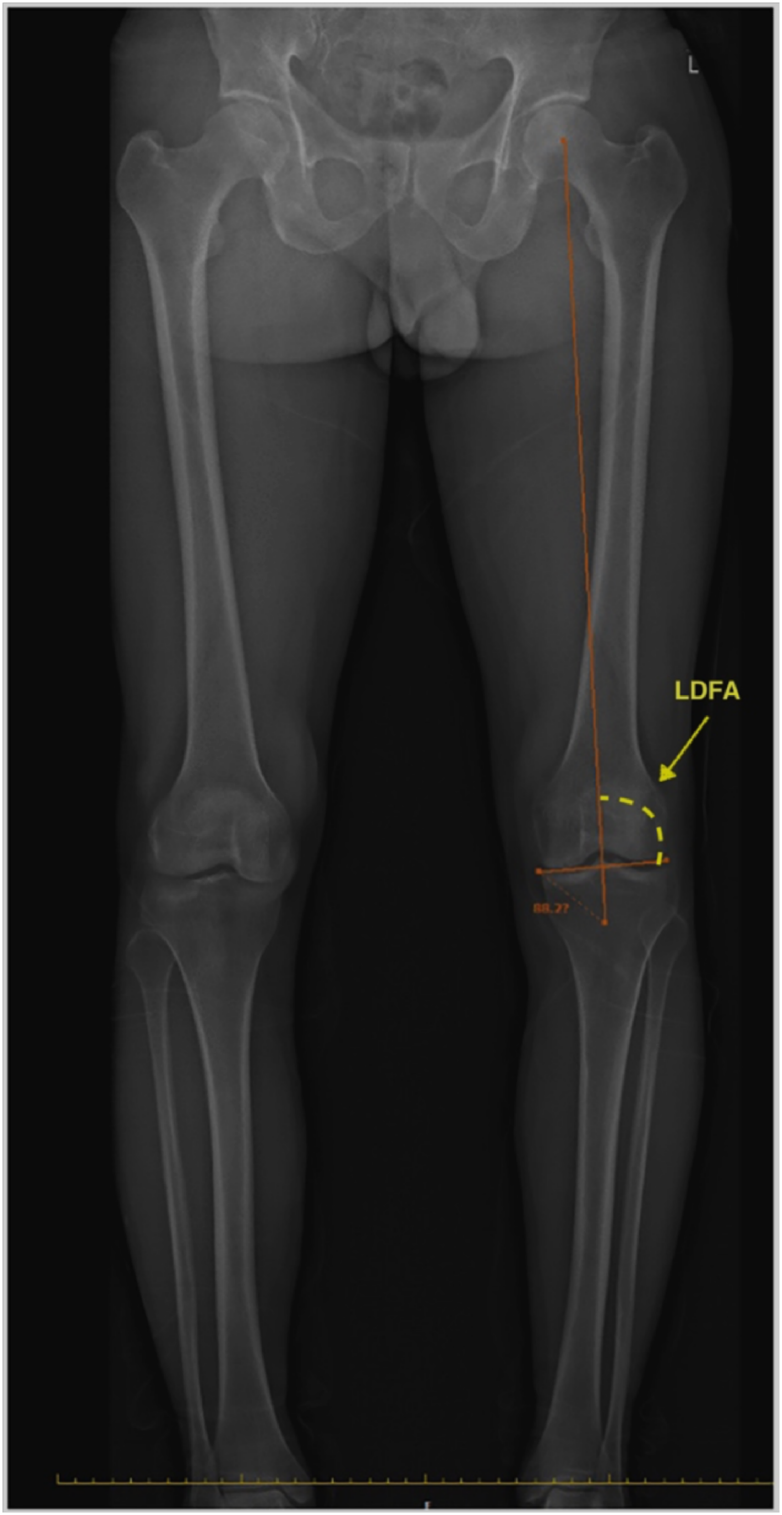
Lateral distal femoral angle (LDFA): the lateral angle formed between the femoral mechanical axis and the knee joint line of the femur in the frontal plane. In this image, the LDFA is 88.2°.

**Figure 4:**
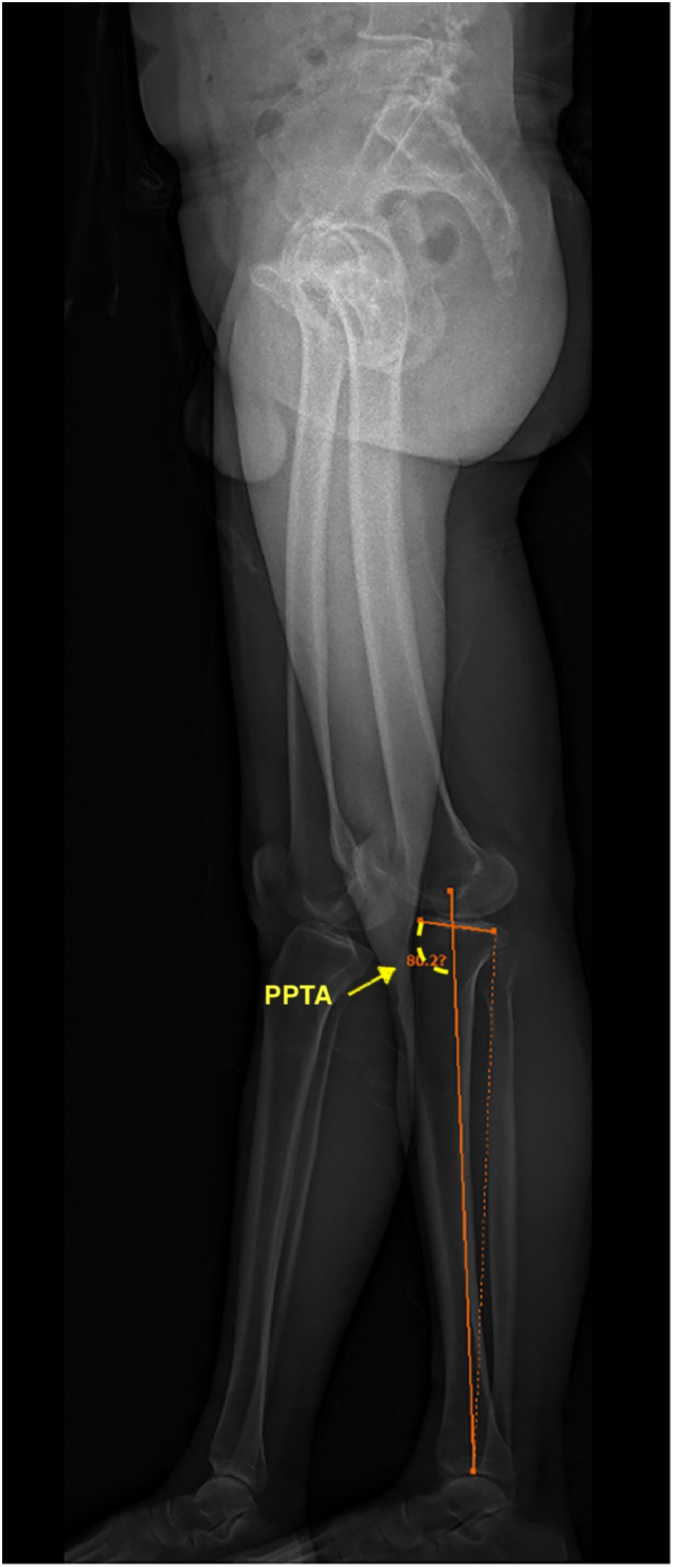
Posterior slope of the proximal tibia angle (PPTA): the angle formed between tibial plateaus and the anatomic axis of the tibia in the sagittal plane. In this image, the PPTA is 80.2°.

**Figure 5:**
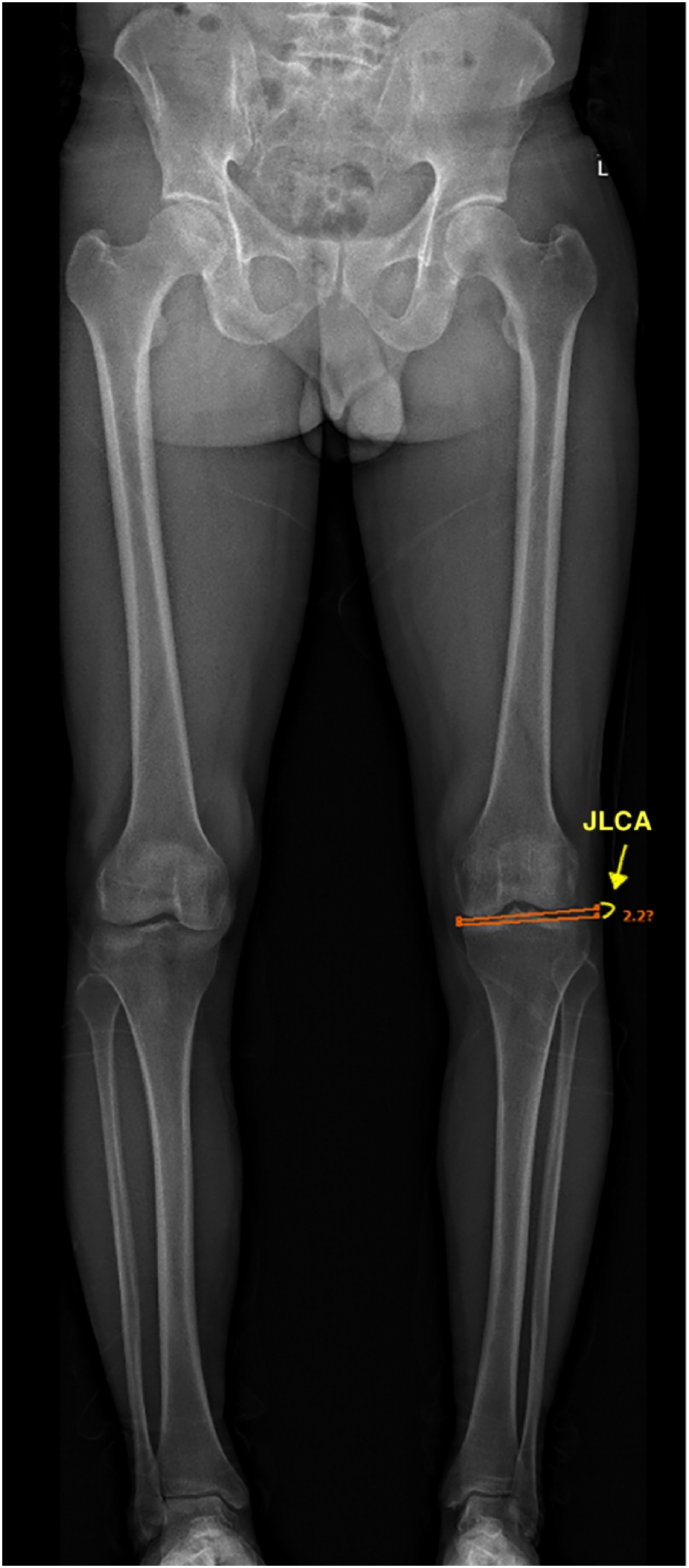
The joint line converge angle (JLCA): the angle between the distal femoral joint line and the proximal tibial joint line in the coronal plane. In this image, the JLCA is 2.2°.

**Figure 6:**
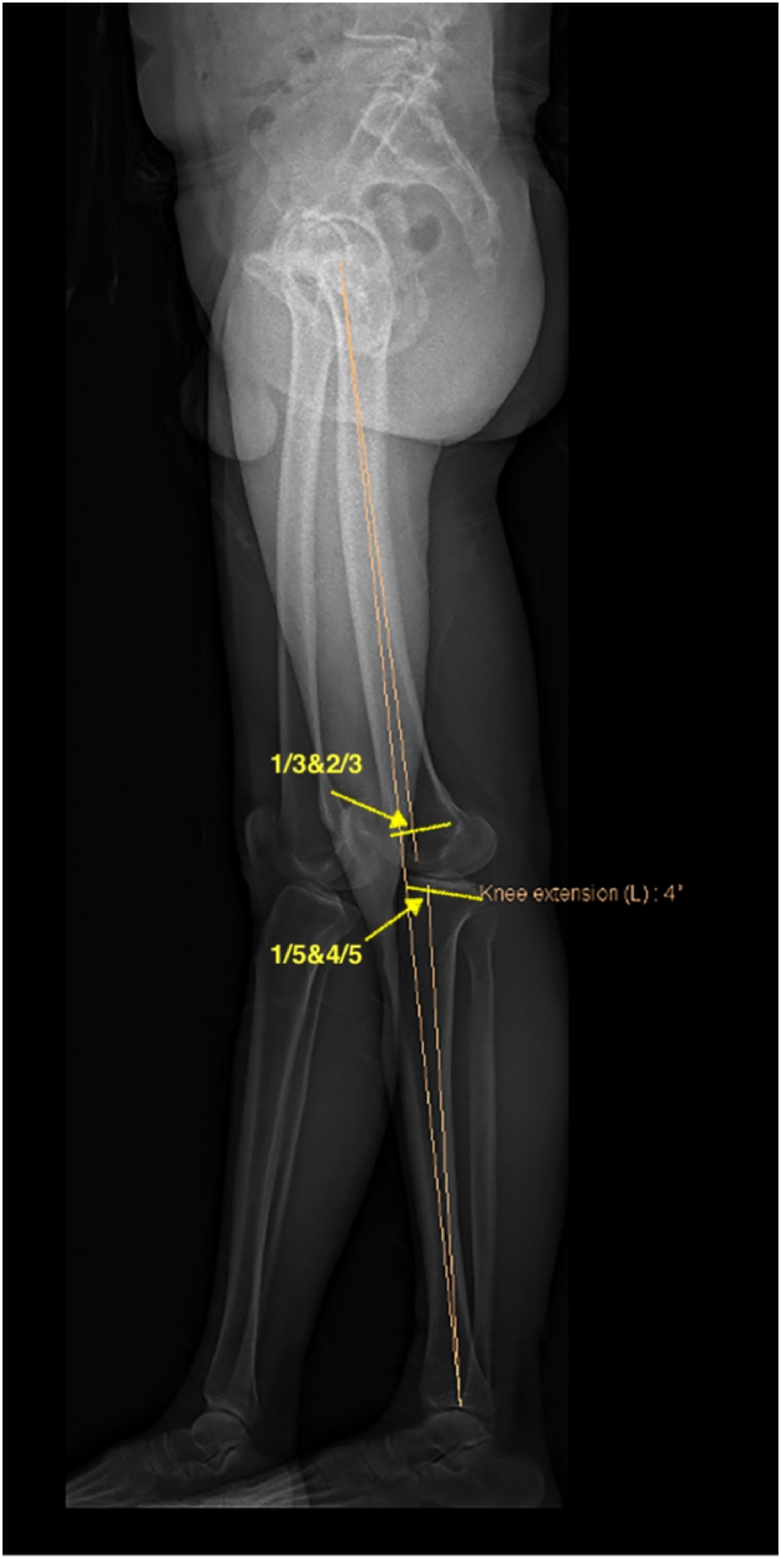
Fixed flexion deformity (FFD): in the sagittal plane, the hip joint center is the center of the femoral head, the center of the distal femur is the junction of the anterior 1/3 and the posterior 2/3 of t trace of the closed femoral growth plate. The mechanical axis of the femur passes through these two points. The proximal tibia center is the junction of the anterior 1/5 and the posterior 4/5 of the tibial plateaus; the ankle joint center is the midpoint of the tibial distal plate. The mechanical axis of the tibia passes through these two points. The angle formed between the femoral mechanical axis and the tibial mechanical axis in the sagittal plane when knees are fully extended. In this image, the FFD is −4°.

**Figure 7:**
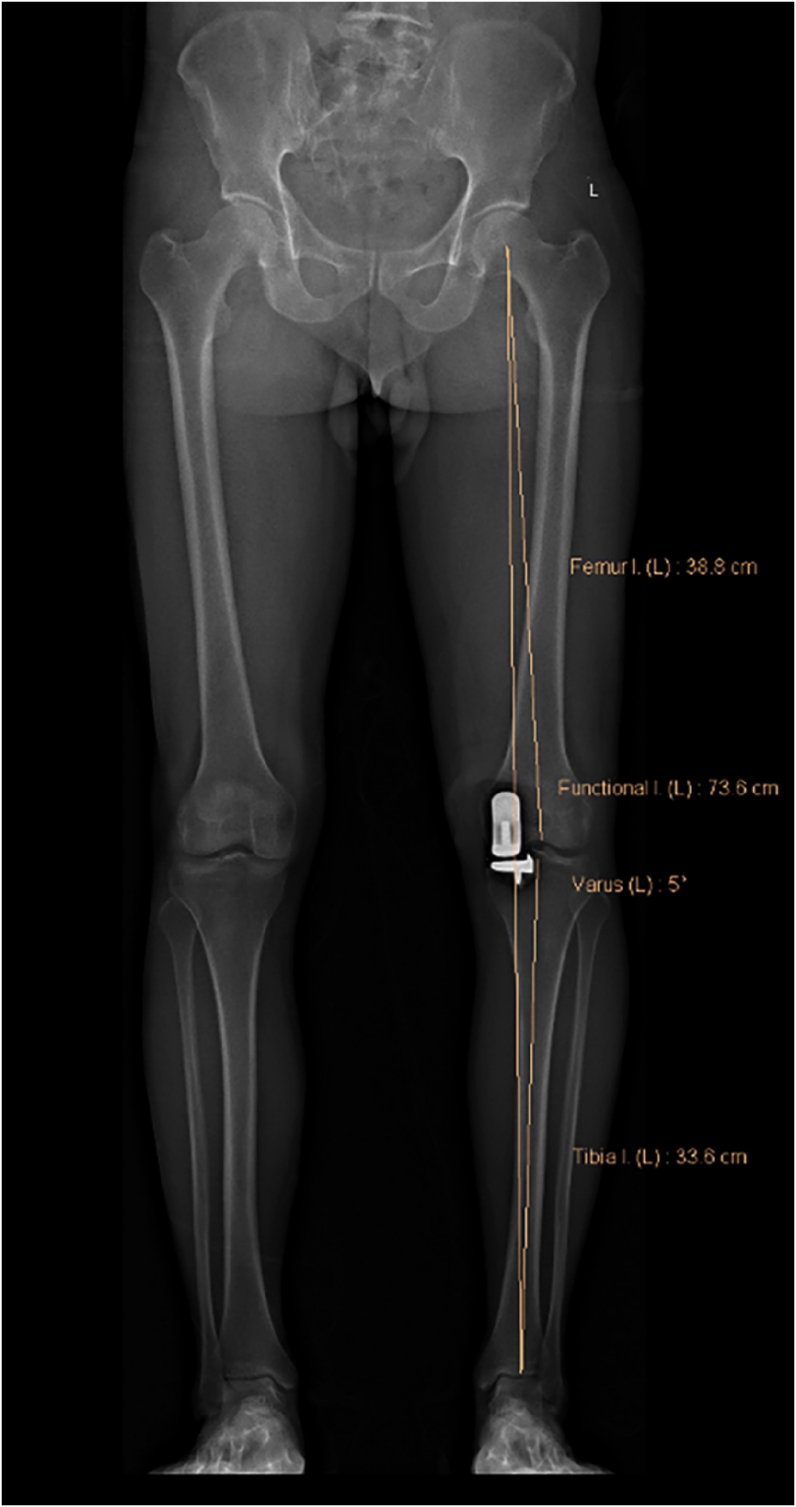
Post-operative noyes angle. It is designated as “varus” in EOS software. In this image, the Noyes angle is 5°.

Anteroposterior (AP) and lateral images in standing positions were obtained and reconstructed using EOS Stereos® software (21). Hip-Knee-Ankle standing imaging was obtained as standard workup pre-operatively and post-operatively. Patients were instructed to stand straight with both knees fully extended and evenly distribute their body weight between both limbs. Post-operative radiographs were obtained three months after surgery for the patients who could follow the instructions and orient themselves in the correct position. Two assessors performed the radiographic assessment according to the validated methods used by Paley (22). The following angles were measured (Figures 1–7): (1) pre-operative Noyes angle: the angle between the femoral mechanical axis (center of the hip to the intercondylar notch of the knee) and the tibial mechanical axis (center of tibial spines to center of the distal tibia); (2) Medial Proximal Tibial Angle (MPTA): the proximal medial angle formed between the tibial mechanical axis and the knee joint line of the tibia in the coronal plane; (3) pre-operative Lateral Distal Femoral Angle (LDFA): the lateral angle formed between the femoral mechanical axis and the knee joint line of the femur in the coronal plane; (4) Posterior slope of the Proximal Tibia Angle (PPTA): the angle formed between tibial plateaus and the anatomic axis of the tibia in the sagittal plane; (5) Joint Line Converge Angle (JLCA): the angle formed between femoral and tibial joint orientation line in the coronal plane; (6) Fixed Flexion Deformity (FFD): the angle formed between the femoral mechanical axis and the tibial mechanical axis when knees are fully extended in the sagittal plane; the mechanical axis of femur was determined by (7) post-operative Noyes angle. Two technicians took these measurements, everyone measured two times, and the average value was recorded.

All cases were divided into either the corrected mechanical axis group (varus ≤5°) or the under-corrected mechanical axis group (varus >5°), according to the degree of post-operative varus (as described in Noyes et al*.*). There were 875 knees in the corrected group and 127 in the under-corrected group.

### Statistical analysis

Pearson chi-square was used to analyze categorical data such as gender and surgical side. Mann-Whitney U was used to analyze continuous data such as age, BMI, ROM, AKSS score, MPTA, LDFA, JLCA, and FFD. SPSS 26.0 (IBM, Chicago, United States) software was employed for statistical analyses. Descriptive analyses were reported using means and standard deviations (SD) for continuous variables and frequencies with percentages for discrete variables. Comparison of the reliability of two measurers' data by calculating Intraclass correlation. Logistic regression is used to estimate the association of one or more independent (predictor) variables with a binary dependent (outcome) variable. A *P* value <0.05 was considered statistically significant.

## Results

### Comparison of pre-operative data

The Noyes method was used to measure the degree of varus in the patient. Patients were subsequently divided into two groups: (1) the corrected mechanical axis group (varus ≤5°) and (2) the under-corrected mechanical axis group (varus >5°). The demographic characteristics of the patients and pre-operative data of the two groups are shown in Table 1.

**Table 1:** Demographic information and a pre-operative score of patients.

Items	Corrected group	Under-corrected group	*χ*2/z	*p*-value
Knees	875	127	/	/
Sex (M/F)	170/583	31/96	0.629	0.428
Side (L/R)	417/458	61/66	0.006	0.937
Age (year) BMI Clinical AKSS Function AKSS ROM (°)	64.7 ± 8.0 26.8 ± 3.5 56.5 ± 14.4 65.3 ± 11.5 105.7 ± 21.2	64.40 ± 7.5 26.1 ± 3.1 55.5 ± 14.2 63.6 ± 11.6 97.4 ± 16.8	−0.188 −1.855 −1.265 −1.645 −3.661	0.851 0.064 0.206 0.100 <0.001*

Values are mean ± SD.

* *p *< 0.05.

The mean pre-operative knee varus angle of all patients was 6.3° ± 2.8°, while the mean post-operative varus angle was 3.8° ± 1.3°. The post-operative varus significantly improved compared to the pre-operative varus (*p* < 0.05).

There were 875 knees in the corrected group and 127 in the under-corrected group. The pre-operative and post-operative varus of the corrected group were 6.0° ± 2.7° and 3.4° ± 1.0°, respectively. The pre-operative and post-operative varus of the under-corrected group were 8.2° ± 2.9° and 6.4° ± 0.8°, respectively. Post-operative varus was significantly improved in both groups (*p* < 0.05). However, no differences were observed in gender, surgical side, age, BMI, pre-operative ROM, pre-operative clinical AKSS, and function AKSS when comparing the two groups, as shown in Table 1.

The pre-operative x-ray parameter measurements of the two groups are shown in Table 2. Pre-operative Noyes angle, MPTA, and FFD significantly differed between the two groups (*p* < 0.05). The pre-operative Noyes angles of the corrected and under-corrected groups were 7.0° ± 2.7° and 9.2° ± 2.9°, respectively. The pre-operative MPTA in the corrected group and the under-corrected group was 85.7° ± 1.4° and 84.2° ± 1.6°, respectively. Finally, the pre-operative FFD in the corrected and under-corrected groups were 5.3° ± 5.5° and 7.4° ± 5.2°, respectively. No significant difference was observed between the two groups pre-operatively in JLCA, LDFA, and PPTA.

**Table 2:** Pre-operative measurement results of corrected and under-corrected groups.

Items	Corrected (*n* = 875)	Under-corrected (*n* = 127)	z	*p*-value
Noyes (°)	7.0 ± 2.7	9.2 ± 2.9	−7.929	<0.001*
MPTA (°)	85.7 ± 1.4	84.2 ± 1.6	−10.412	<0.001*
LDFA (°) JLCA (°)	88.7 ± 1.3 4.2 ± 1.9	88.7 ± 1.3 4.8 ± 1.6	−0.55 −15.828	0.957 <0.001
PPTA (°)	81.3 ± 2.4	81.3 ± 2.4	−0.365	0.715
FFD (°)	5.3 ± 5.5	7.4 ± 5.2	−4.438	<0.001*

Values are Mean ± SD.

*p < 0.05 denotes a significant difference.

### Logistic regression analysis of pre-operative risk factors for varus after UKA

Pre-operative Noyes angle >10°, LDFA >90°, MPTA <85°, JLCA >5°, PPTA >82°, FFD >10° were determined to be independent variables (Table 3), based on whether post-operative varus was >5°. As we know, LDFA, MPTA, and JLCA are essential parameters to describe the coronal features of the knee joint, and PPTA is an important parameter to describe the sagittal features of the knee joint. According to Paley (22), the normal LDFA = 88°, MPTA = 87°, JLCA = 2∼3°, and PPTA = 80°. Therefore, we set the deviation ≥3°from the normal as a variable for analysis. Noyes angle is the most intuitive parameter to describe the coronal force line of the knee joint. According to Goodfellow (12), AMOA is mostly 5∼15° varus, and the anterior cruciate ligament failure is considered when the varus is greater than 15°; Oxford UKA is not recommended.FFD is the most visualized parameter to describe the sagittal force line of the knee joint. Goodfellow suggested that if FFD > 15°, UKA was not recommended. Therefore, we set FFD > 10° as the variable for analysis. We first screened using a one-way chi-square test and found that Noyes >10°, MPTA <85°and FFD >10° were independent risk factors for postoperative varus (Table 4). Subsequently, logistic multivariate regression analyses were performed on these variables in the under-corrected group. Our findings showed that the pre-operative MPTA (*p* < 0.001, OR=4.522, 95% CI: 2.927–6.984), pre-operative Noyes angle (*p* < 0.001, OR=3.262, 95% CI: 1.802–5.907), and pre-operative FFD (*p* = 0.007, OR=1.862, 95% CI:1.182–2.934) were independent risk factors associated with post-operative residual (Table 5).

**Table 3:** The set of independent variables in multivariate analysis.

Variable			Corrected		Under-corrected	
		Case (*n*)		Percentage (%)	Cases (*n*)	Percentage (%)
Noyes >10°	Yes	79		9.1	46	36.2
	No	796		90.9	81	63.8
MPTA <85°	Yes	191		21.8	81	63.8
	No	684		78.2	46	36.2
LDFA >90°	Yes	120		13.7	19	12.5
	No	755		86.3	108	87.5
JLCA >5°	Yes	458		52.3	51	40.2
	No	417		47.7	76	59.8
PPTA >82°	Yes	474		54.2	71	55.9
	No	401		45.8	56	44.1
FFD >10°	Yes	162		18.5	38	29.9
	No	713		81.5	89	70.1

**Table 4:** Univariable analysis of pre-operative factors associated with varus after UKA.

Variables	*χ*2	*p*-value
Noyes >10°	75.102	<0.001
MPTA <85°	98.690	<0.001
LDFA >90°	0.144	0.704
JLCA >5°	6.589	0.010
PPTA >82°	0.134	0.714
FFD >10°	157.060	<0.001

**Table 5:** Logistic regression analysis of pre-operative risk factors for varus after UKA.

Variable	B	Wald	OR	95% CI	*p*-value
Noyes >10°	1.182	15.241	3.262	1.802–5.907	<0.001*
MPTA <85°	1.509	46.270	4.522	2.927–6.984	<0.001*
LDFA >90°	−0.365	1.351	0.700	0.384–1.277	0.245
JLCA >5°	−0.019	0.005	0.981	0.591–1.630	0.942
PPTA >82°	0.044	0.044	1.045	0.696–1.569	0.834
FFD >10°	0.622	7.183	1.862	1.182–2.934	0.007*

* Denotes statistical significance.

## Discussion

Alignment of the lower extremity after UKA was crucial (15). It has been reported that over-correction easily causes cartilage degeneration and even OA of the lateral compartment, which leads to the overall failure of the UKA (24).To avoid post-operative valgus, a slight under-correction is considered acceptable for UKA and TKA. However, severe under-correction can increase the risk of severe polyethylene wear and aseptic loosening (24–26). Finite elemental analysis showed that the stress of the medial compartment would be significantly increased due to the varus of the post-operative mechanical axis. As this can be a point of pain for the patient (27), it is essential to avoid severe varus after UKA. Scholars have different views on the ideal alignment after UKA. Goodfellow (12) and Mullaji et al. (28) believed that the alignment of the lower extremity after unicondylar replacement should be restored to the position before osteoarthritis. Kim et al. (29) found that the alignment of the lower extremity after unicondylar replacement mainly depends on the thickness of the polyethylene insert and has nothing to do with the placement of the femoral condyle and tibial prosthesis.

Contrary to the findings of Zhang et al. (30), they believed that there was no significant relationship between the alignment of the lower extremity and the thickness of the spacer after unicondylar replacement. It mainly depended on the position of the alignment of the lower extremity before surgery and the amount of osteotomy during the operation. Based on former findings, most surgeons advocate for a minor post-operative residual varus in medial UKA patients (8, 15, 31). Many studies indicate that maintaining a mild varus of the post-operative mechanical axis could improve survivorship (32). Furthermore, a minor varus of the mechanical axis does not compromise the outcome of a medial UKA and yields better results than a neutral or close-to-neutral mechanical axis (8).

This study is the first to report pre-operative factors that affect varus after Oxford UKA to the best of our knowledge. We believe a post-operative lower limb alignment of 1°-5° varus should be pursued, as it can provide more relief for the patient. Chatellard had the same viewpoint (15). Kim followed up on 246 UKA cases and concluded that patients with mild varus postoperatively had the highest prosthesis survival rate (33). Vasso and Zuiderbaan also reported significantly higher post-operative outcome scores (International Knee Society and Western Ontario and McMaster Universities Osteoarthritis Index, respectively) in patients with a post-operative varus of 4° on the mechanical axis (8, 9). Taking these studies into account, it should be noted that minor varus alignment of <5° after medial UKA is considered “corrected,” while > 5° varus alignments are categorized as “under-corrected.” In our study, the surgical goal was to achieve a post-operative lower limb alignment of 1°- 5° varus.

This study divided all cases into two groups by the extent of post-operative varus. The corrected group included patients whose residual varus was < 5°, while the under-corrected group included patients whose residual varus were > 5°. Of the patients participating, 12.7% had some form of varus after UKA. There were multiple reasons for the post-operative varus. This study analyzed the influence of pre-operative factors on post-operative varus. By multivariate analysis, the independent influencing factors that significantly impacted post-operative varus were as follows: (1) pre-operative MPTA (*p* < 0.001, OR=4.522, 95% CI: 2.927–6.984), (2) pre-operative Noyes angle (*p* < 0.001, OR=3.262, 95% CI: 1.802–5.907) and (3) pre-operative FFD (*p* = 0.007, OR=1.862, 95% CI: 1.182–2.934). The effects of pre-operative LDFA (*p* = 0.146), JLCA (*p* = 0.942), and pre-operative PPTA (*p* = 0.899) did not show statistically significant results on the postoperative mechanical axis.

Pre-operative Noyes >10° indicated that the patient had a pre-operative mechanical axis with severe varus, and there might be a certain extent of medial collateral ligament contracture. Per Oxford's recommendations, extensive MCL release was avoided as much as possible to prevent serious complications such as medial instability, post-operative mechanical axis valgus, and even polyethylene insert dislocation (25, 34). The contracture of the medial structure made the filling amount of the medial gap smaller, so it was more prone to be varus after surgery. MPTA and LDFA reflected the extent of the extra-articular deformity as the first step of Oxford UKA is performing an osteotomy on the tibial side. The osteotomy of the posterior femoral condyle and the distal femur was determined by referring to the osteotomy plane of the tibia. Therefore, choosing the tibial osteotomy plane is an essential step in the process of Oxford UKA. If the MPTA was less, the proximal tibia varus was more severe, and the tibial plateau displayed a more significant tilt. An intersection angle of more than 10° between the medial tibia plateau's osteotomy line and the lateral plateau's joint line should be avoided when performing the tibial osteotomy, as it is necessary to keep the orientation of the medial platform and the lateral platform consistent. For patients whose MPTA was severely abnormal, the angle of the osteotomy of the medial tibial plateau should be placed in the minor varus so that the intersection angle is not too great. The tibia remained in minor varus inevitably. According to the results of this study, patients with MPTA <85° may have a 4.5 times chance of post-operative residual varus compared to patients with MPTA ≥85° pre-operatively. The smaller MPTA was an essential factor that led to residual varus after surgery. Moreover, when there is a significantly lower predicted probability of achieving optimal post-operative alignment, other treatments, such as high tibial osteotomy, may be considered in this patient.

A large pre-operative FFD of >10° indicates post-operative residual varus. The difference in pre-operative FFD between the two groups may lead to the difference in pre-operative ROM between the two groups. Long-term FFD may result in the contracture of the medial posterior corner of the knee joint, which tightens the medial extension gap, resulting in more bone grinding in the distal femur. Pre-operative LDFA did not broadly affect post-operative varus because the femur of Oxford UKA refers to the orientation of the tibia joint line, which is used to balance flexion and extension. The amount of bone removed from the distal femur is not determined by the anatomy of the femur itself. PPTA reflects the posterior tibia slope angle (PTSA) and may affect tibial osteotomy, so we included it in the study. However, pre-operative PPTA did not affect the mechanical axis correction. So, the posterior tibia slope does not appear to affect the post-operative mechanical axis.

In the case of medial OA, medial JLCA convergence is often due to medial cartilage loss. JLCA, as a parameter reflecting the degree of intra-articular deformity, is caused by the loss of the cartilage layer of the degenerated joint. The lower limb mechanical axis after medial UKA is driven primarily by the correction of the joint line deformity in these patients. This was based on the rationale that medial UKA restores the joint line height and improves joint congruence, as was shown by Chatellard and Khamaisy (15, 35). By restoring the joint line height and congruence within the knee joint. When the medial compartment is reconstructed after UKA, the varus caused by JLCA is eliminated, so it has no impact on the post-operative varus.

Previously, the valgus stress view radiograph has been utilized to ascertain the ability to correct the deformity, ensure maintenance of the lateral joint space, and indirectly assess the integrity of the anterior cruciate and medial collateral ligaments. It is often discussed as an essential criterion for determining a patient's candidacy for medial UKA. Proponents of the valgus stress x-ray often state that it must demonstrate “full correctability” of the deformity without narrowing the lateral joint space for a patient to be considered a candidate for medial UKA (32). However, Tyler's research (36) showed that pre-operative stress radiographs overstated value in patients undergoing medial UKA since the full extent of correctability of the varus deformity cannot be determined until after removing osteophytes and as most deformities are not fully correctable to neutral in UKA. The significance of our study is to elucidate the relevant factors of lower limb mechanical axis recovery after UKA. The recovery of the post-operative mechanical axis can be determined from the pre-operative x-ray film. Tashiro (32) reported that the post-operative lower limb mechanical axis is highly correlated with the pre-operative valgus stress view radiograph and moderately correlated with the orthotopic position before and after the surgery. x-ray under varus or valgus stress is more difficult to obtain as there is no unified standard for measurement. All the pre-operative measurement parameters selected in this study are routinely obtained before UKA, so no additional measures are needed.

According to the results of this study, if the pre-operative varus deformity is mainly from the tibial side, the possibility of residual varus after the operation is relatively high. For example, patients with FFD are also more prone to post-operative varus. Femoral deformities had no effect on residual varus after surgery. It was likely to be corrected post-operatively if intra-articular deformities contributed to the varus deformity. Surgeons can predict the risk of postoperative mechanical axis varus *via* routine pre-operative measurements. Oxford does not recommend extensive release of the MCL to avoid dislocation of the insert. Goodfellow pointed out (12) that the medial collateral ligament should not be released during oxford UKA surgery to prevent postoperative valgus or dislocation of the polyethylene insert. In our operation, we hope that the patient's postoperative alignment can be maintained in varus 1–5°. Therefore, a slight adjustment of medial support structure tension may be required in some cases with severe medial support structure contracture and varus. For patients with a high risk of postoperative varus, consideration should be given to reducing the amount of tibial osteotomy or using a thicker insert to correct varus. If the medial tension is too large, removal of medial osteophytes and dissection of the adhesions between the medial support structure and the proximal tibia can be considered for release. But try not to carry out the direct release of the MCL body and the insertion to ensure MCL is intact. The role of soft-tissue balancing in correcting the lower limb mechanical axis after UKA could be assessed in future studies. A previous TKA study reported that a tight soft-tissue envelope in patients with a varus deformity >10° could contribute to bony deformity (37). Particularly for patients with less MPTA, surgeons need to focus on the amount of osteotomy. A 1 mm spoon or the 3 mm G-type clip was used to reduce tibial osteotomy and correct the mechanical axis by filling the medial compartment.

The limitation of this study is that there is no follow-up of clinical results. However, evaluating clinical function is not the purpose of this study. Further research analyzes the relationship between lower limb mechanical axis changes and the patient's clinical function.

## Data Availability

The original contributions presented in the study are included in the article/Supplementary Material, further inquiries can be directed to the corresponding author/s.
